# Design and development of adapters for electromagnetic trackers to perform navigated laparoscopic radiofrequency ablation

**DOI:** 10.1186/1750-1164-1-7

**Published:** 2007-10-31

**Authors:** Philipp Hildebrand, Armin Besirevic, Markus Kleemann, Stefan Schlichting, Volker Martens, Achim Schweikard, Hans-Peter Bruch

**Affiliations:** 1 University of Schleswig-Holstein, Campus Lübeck, Department of Surgery, Ratzeburger Allee 160, Lübeck, 23538, Germany; 2 University of Lübeck, Institute for Robotics and Cognitive Systems,, Ratzeburger Allee 160, Lübeck, 23538, Germany

## Abstract

**Background:**

Laparoscopic radiofrequency ablation (RFA) is an accepted approach to treat unresectable liver tumours distinguishing itself from other techniques by combining minimal invasiveness and the advantages of a surgical approach. The major task of laparoscopic RFA is the accurate needle placement to achieve complete tumour ablation. The use of an ultrasound-based, laparoscopic online-navigation system could increase the safety and accuracy of punctures. To connect such a system with the laparoscopic ultrasound (LUS) transducer or the RFA needle especially designed adapters are needed. In this article we present our first experiences and prototypes for different sterilizable adapters for an electromagnetic navigation system for laparoscopic RFA.

**Methods:**

All adapters were constructed with the help of a standard 3D CAD software. The adapters were built from medical stainless steel alloys and polyetherketone (PEEK). Prototypes were built in aluminium and polyoxymethilen (POM). We have designed and developed several adapters for the connection of electromagnetical tracking systems with different RFA needles and a laparoscopic ultrasound transducers.

**Results:**

Based on earlier experiences of the initial version of the adapter, sterilisable adapters have been developed using biocompatible materials only. After short introduction, the adapters could be mounted to the laparoscopic ultrasound probe and the RFA needle under sterile conditions without any difficulties. Laboratory tests showed no disturbance of laparoscopic navigation system by the adapters. Anatomic landmarks in the liver could be safely reached. The adapters showed good feasibility, ergonomics, sterilizability and stability.

**Conclusion:**

The development of usable adapters is the prerequisite for accurate tracking of a RFA needle for laparoscopic navigation purposes as well as 3D navigated ultrasound data acquisition. We designed, tested and used different adapters for the use of a laparoscopic navigation system for the improvement of laparoscopic RFA.

## Background

Laparoscopic Radiofrequency-ablation is a save and effective method for local tumour destruction and offers a combination of minimal invasive surgery and the advantages of laparotomy. However in contrary to the transcutaneously free-hand puncture application of the laparoscopic free-hand puncture is restricted because of the capnoperitoneum and the consecutive fixation of the needle on 2 different points. The use of a laparoscopic ultrasound probe with a canal for puncture can solve this problem and improve the precision of puncture. However, a stiff needle limits the necessary angulation that is needed to reach right-lateral and cranial liver metastases [1].

An ultrasound-based, laparoscopic online-navigation system, which allows guided out-of-plane needle placement, could significantly increase the accuracy of puncture. The system is aligned to help the surgeon to perform laparoscopic radiofrequency ablations using preoperative planning data and tracked laparoscopic ultrasound (LUS) as well as a tracked RFA needle [2]. The tracking hardware for the prototype of the system consists of an electromagnetical tracking system. In order to connect the tracking system with the RFA needle and the LUS, different adapters have to be used. These adapters should provide a reproducible, sterilisable, easy to handle and save connection between the trackers and the RFA needle and the LUS respectively. Furthermore, the adapters need to be designed in a way that the electromagnetic tracking system is not influenced. In this article, we present our experiences and solutions with the development of electromagnetic adapters.

## Methods

### First experiences

#### Adapter for laparoscopic ultrasound probe

**Figure 1:**
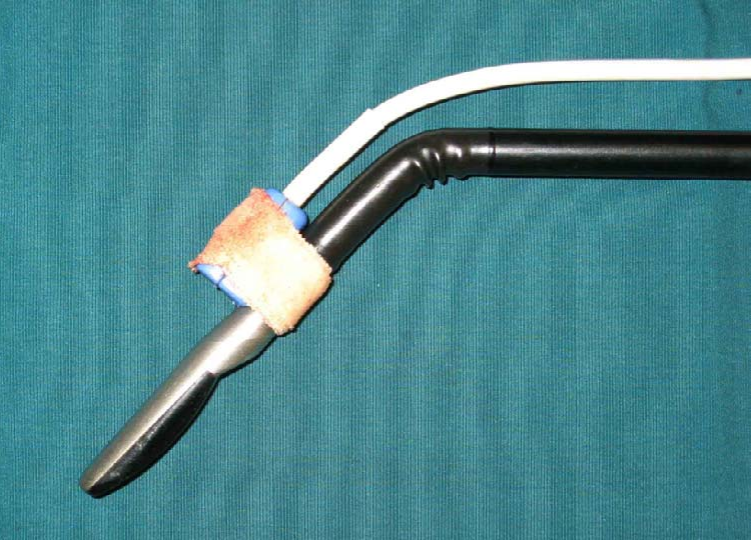
Connection of laparoscopic ultrasound transducers with electromagnetic tracking system US Guide 2000^® ^with adhesive tape.

**Figure 2:**
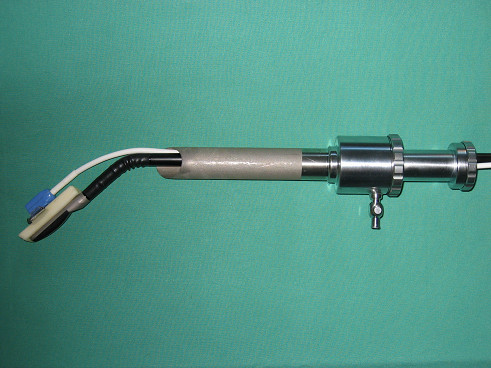
LUS connected to the navigation system US Guide 2000^® ^inserted through a 22 mm trocar in an experimental environment.

**Figure 3:**
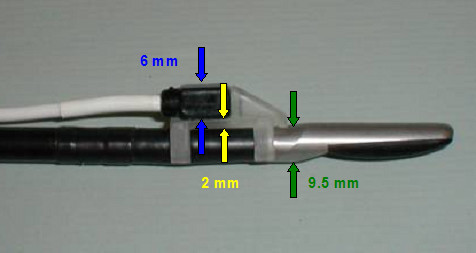
Adapter with mounted tracking sensor from the US Guide 2000^® ^at the head of the LUS with a total diameter of 17,5 mm.

In a first step, the electromagnetic tracker of the US-Guide 2000™ system (Ultra Guide^®^, Tirat Hacarmel, Israel), which is a six-degree-of-freedom electromagnetic tracking system for percutaneous interventions, was connected to the LUS transducer (B-K Medical Denmark, Type 8815) by using adhesive tape. The nearer the adapter could be placed to the tip of the instrument, the higher is the accuracy of the system. The tracking of the LUS was feasible but too inaccurate (Fig 1). Therefore a tighter and more stable adapter was developed in order to solve this problem. This adapter was made of Acrylnitrit/Butadien/Styrol-Copolymer ABS and shaped to fit the convexity of the ultrasound probes head. The LUS with the connected adapter could be placed through a 22 mm trocar without any problems (Fig. 2). However, treatments with this adapter could not be performed under sterile conditions. Therefore the development was focused on sterilisable and biocompatible materials. By doing this, it was possible to design sterilisable adapters for the LUS transducer. However, the adapter with the mounted tracking sensor at the head of the laparoscopic ultrasound probe had a total diameter of 17,5 mm, which still made a 22 mm trocar necessary (Fig. 3) [2-4].

#### Adapter for laparoscopic RFA needle

**Figure 4:**
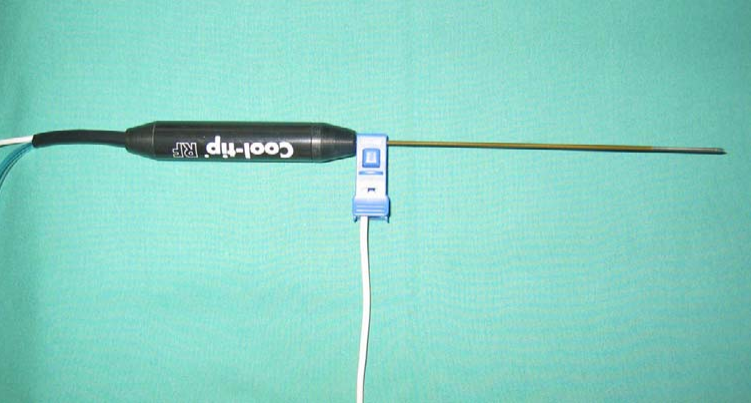
Adapter with mounted tracking sensor from the US Guide 2000^® ^at the needle of the radiofrequency electrode.

For the connection of the US-Guide 2000™ navigation system to either expandable RFA needles (RITA Medical System Inc., Mountain View, CA, USA) or internally cooled RFA needles (Radionics Inc., Burlington, MA, USA) the electromagnetic tracker was mounted to the basis of the RFA needle (Fig. 4). We have used the adapter from the US-Guide 2000™ navigation system, which is normally designed for transcutaneous guided punctures and connected with the RFA needle in perpendicular direction. However, rotations of the adapter around the needle could not be compensated by the navigation system.

### Newly developed adapters

All newly developed adapters were constructed using standard 3D CAD software (Solid Edge, UGS). The adapters were build from medical stainless steel alloys (1.4301 or 1.405) and Polyetherketone (PEEK). Prototypes were built in aluminium and polyoxymethilen (POM).

Furthermore, new electromagnetic tracking systems (NDI Canada, Type AURORA^® ^and Ascension, Type 3D-Guidance^®^) with smaller electromagnetic trackers were used.

#### New adapters for laparoscopic ultrasound probe

**Figure 5:**
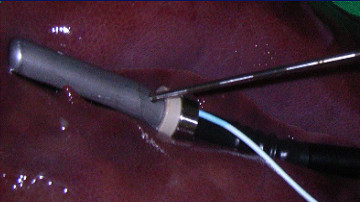
Connection of LUS transducers with AURORA^® ^electromagnetic tracking by the "ring" adapter.

**Figure 6:**
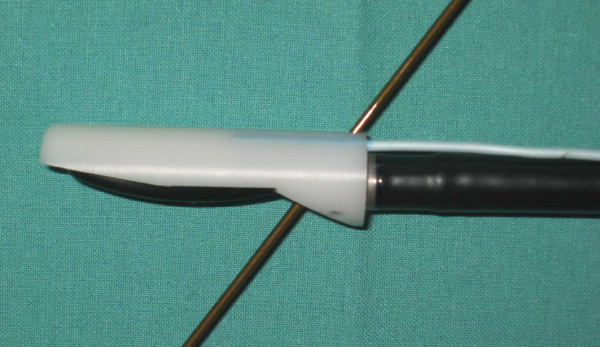
Connection of the LUS transducer with the AURORA^® ^electromagnetic tracking system by using the "shoe" adapter.

**Figure 7:**
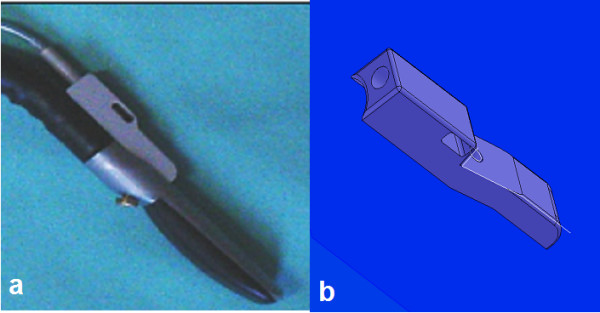
Connection of the LUS transducer with any electromagnetic tracking system offering sensors with a diameter below 1.8 mm (a) and presentation of the technichal drawings from the 3D CAD software (b)

We first designed two different adapters to combine the LUS transducer with the electromagnetic tracking system. The first LUS adapter is a ring, which consists of two materials (PEEK and stainless steel alloys 1.405), that can be placed directly on the flexible part of the probe. The sensor is placed inside the inner PEEK part and is fixed with the outer steel part. It was evaluated in combination with the image-guided navigation system. The experimental setup consists of a perfused tumor-mimic-model of the porcine liver (Fig. 5) [5,6]. The second adapter, which is made of polyoxymethilen (POM), is pulled over the tip of the probe like a shoe (Fig. 6). The sensor is placed in a drilled hole at the back part of the adapter. Both adapters fit through a 17 mm trocar and were investigated regarding its influence on the electromagnetic trackers. Most recently we were able to design an adapter, which can be used with a 15 mm trocar and the 3D-Guidance tracking system (Fig. 7). This one is also made of PEEK and is mounted to the LUS tip using the puncture channel. Due to sensitivity of the electromagnetic sensors it is fixed inside a PEEK cover that shields the sensor. This Cover is also used to connect the sensor to the back part of the adapter in reproducible way.

#### New adapter for laparoscopic RFA needle

**Figure 8:**
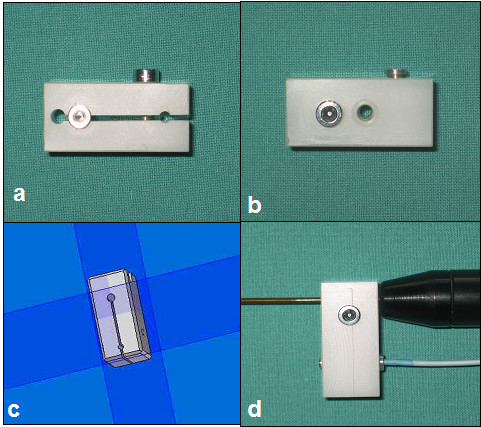
Special developed adapter for the laparoscopic RFA-probe (a + b), which is mounted to the basis of the RFA-needle (d) and presentation of the technichal drawings from the 3D CAD software (c).

The newly designed RFA needle adapter can be mounted on the needle itself (Fig. 8). Due to the rotational symmetry of the needle, only the distance from the needle tip must stay constant during calibration and intra-operative use of the tracked needle. The adapter is connected to the RFA-needle by fixing it with a little screw. The two holes of the adapter are used to take up the needle and the sensor. Like the LUS sensor, this sensor is also fixed inside a PEEK cover.

## Results

The first experiences with the development of adapters for electromagnetic trackers revealed the feasibility of the combination of an electromagnetic navigation system with a RFA needle and LUS [2-4,6]. However, problems of sterilisation, size of the adapters and fixation of the adapters had to be solved. Using the experiences of the initial versions of the adapters, sterilizable adapters have been developed using biocompatible materials only. The new adapters could be sterilized repeatedly without any problems or surface variations. After a short introduction, a surgical nurse and a surgeon were able to mount the adapters to the LUS probe and the RFA needle under sterile conditions without any difficulties.

The adapters for the LUS can be used to acquire navigated three-dimensional ultrasound even from segments that are difficult to reach (right lateral and cranial segments), because the electromagnetic tracker was attached to the tip of the LUS probe, which allows free angulation of the ultrasound transducer without interference of the navigation system. Initial material testing for the ring adapter showed no detectable interferences of the navigation system [7]. New laboratory tests could confirm that there is no influence of the ring adapter on the tracking system. However, the calibration of the ultrasound probe with the tracking system has to be performed after mounting of the ring adapter, which means under sterile conditions in the operating room. This procedure is time-consuming and is not effective for surgical procedures. The shoe-alike adapter prototype for the laparoscopic probe, which allows a reproducible positioning of the sensor, was designed to solve this problem. The shoe adapter, which consists only of POM has no influence on the tracking sensor, too. The most recently developed adapters for the LUS and laparoscopic RFA-needle have also been tested in organ models. They showed no disturbance of the tracking system as well as reproduceble calibration results due to their defined fixation and combined an ergonomic handling with a sufficient stability even after repeated sterilization.

The new developed adapter for the RFA probe showed neither influence on the NDI Aurora nor on the Ascension 3D-Guidance tracking system. Anatomic landmarks in the perfused porcine liver could be safely reached [6,8]. Accurate measurements are still outstanding.

Overall, the newly developed adapter for the LUS and the RFA needle showed good feasibility, ergonomics, sterilizability and stability.

## Discussion

The laparoscopic approach for radiofrequency ablation, originally described by Siperstein et al. in 1997, offers a minimally invasive procedure in combination with the advantages of an open procedure [9]. Above that laparoscopic RFA can be performed with the same exactness and effectiveness in comparison to the open approach in well-selected patients [10,11]. Even carrying a higher access trauma laparoscopic RFA shows no significantly increased morbidity- or mortality-rates in comparison to the percutaneous probe application, which means a safe and mild treatment for the patient [10,12]. Unfortunately today there are also limitations to the laparoscopic approach based on general contraindications for laparoscopic procedures and the limitations of laparoscopic needle application. Ultrasound-guided navigation tools for laparoscopic RFA offer a new technique for interventional liver therapy. The major advantage is the possibility of out-of-plane needle placement and the combination of flexibility of free-hand type procedures with the accuracy of a biopsy-transducer. This improves the safety and accuracy of punctures and leads to an improvement of quality of the intervention. However, in order to combine an electromagnetic navigation system with the LUS and the laparoscopic RFA needle, special designed adapters are needed to mount the trackers to the instruments. These adapters must be sterilizable and ergonomic, should provide reproducibility and need to be designed in a way that the navigation system is not disturbed.

Our evaluation shows the difficulties in designing adapters that combine all required qualifications. Ongoing development is needed to solve existing problems, guarantee steady progress and achieve acceptable results for clinical use. This study presents the feasibility and usability of special designed adapters for electromagnetic trackers to combine an electromagnetic navigation system with the LUS and laparoscopic RFA needle. This is the prerequisite for navigated laparoscopic RFA to improve the accuracy and results of laparoscopic liver interventions.

In the next steps we are planning to perform animal studies with the presented adapters. Furthermore, we are working on further miniaturization of the laparoscopic adapters to reduce the used trocar size. Besides that, adapters for hybrid tracking (optical and electromagnetical) of LUS and different RFA needles are already under evaluation, but can not be presented today for patent reasons.

## Conclusion

The design and development of adapters for electromagnetic trackers is the prerequisite for an accurate tracking of the RFA needle for laparoscopic navigation purposes as well as 3D navigated ultrasound data acquisition. We designed, built, tested and used different adapters for the use of a laparoscopic navigation system for the improvement of laparoscopic RFA.

## Competing interests

The author(s) declare that they have no competing interests.

## Authors' contributions

All named authors have made substantial contributions to conception, design and carrying out of the study by participating in the design and development as well as the experimental testing of the electromagnetic adapters.
